# Supplementary material for: A Promising Approach to Treat Psoriasis: Inhibiting Cytochrome P450 3A4 Metabolism to Enhance Desoximetasone Therapy
Source: Pharmaceutics. 2023 Jul 25;15(8):2016. doi: 10.3390/pharmaceutics15082016 (PMC10458942; doi:10.3390/pharmaceutics15082016)
Supplement: Supplementary file 1 [file pharmaceutics-15-02016-s001.zip › pharmaceutics-2516458-supplementary.pdf]

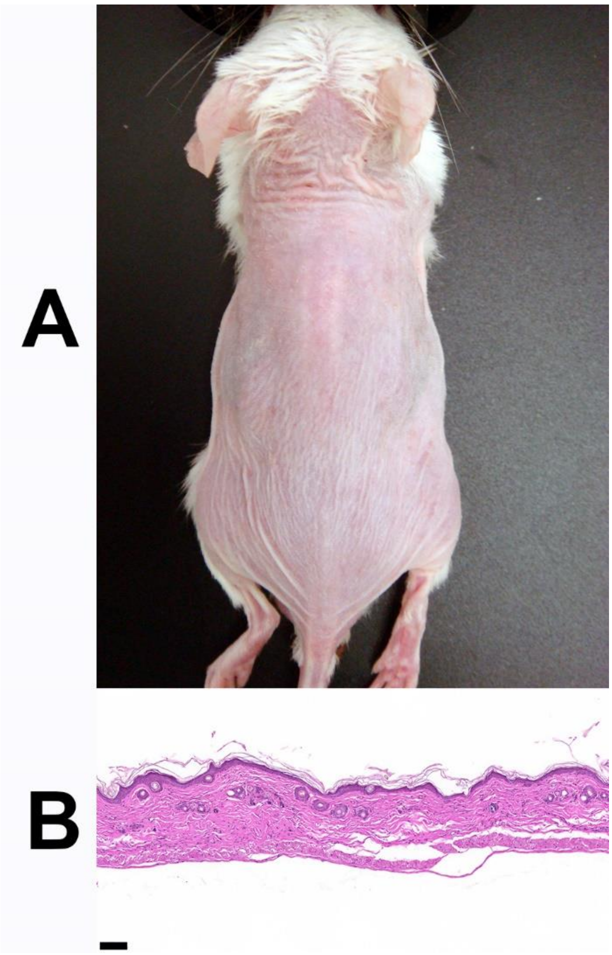

Figure S1. Effects of Clobetasol Treatment on Day 12.

The Clobetasol treatment resulted in a reduction in skin scale and thinner epidermis on Day 12. (A) Morphology (B) H&E staining. Scale bar = 100 μm.
